# Supplementary material for: CD103+ cDC1 and endogenous CD8+ T cells are necessary for improved CD40L-overexpressing CAR T cell antitumor function
Source: Nat Commun. 2020 Dec 2;11:6171. doi: 10.1038/s41467-020-19833-3 (PMC7710757; doi:10.1038/s41467-020-19833-3)
Supplement: Supplementary file 1 — Supplementary Information [file 41467_2020_19833_MOESM1_ESM.pdf]

CD40L-overexpressing CAR T cells induce CD103+ cDC1 to prime endogenous CD8+ T cells for enhanced anti-tumor responses

Nicholas F. Kuhn[1,2,3], Andrea V. Lopez[2], Xinghuo Li[2], Winson Cai[2], Anthony F. Daniyan[2], Renier J. Brentjens[2,*]

[1]Louis V. Gerstner Jr. Graduate School of Biomedical Sciences, Memorial Sloan Kettering Cancer Center, New York, NY, USA.
[2]Department of Medicine, Memorial Sloan Kettering Cancer Center, New York, NY, USA.
[3]Current address: Department of Pathology, University of California San Francisco, San Francisco, CA, USA
*Corresponding author

Supplementary Information

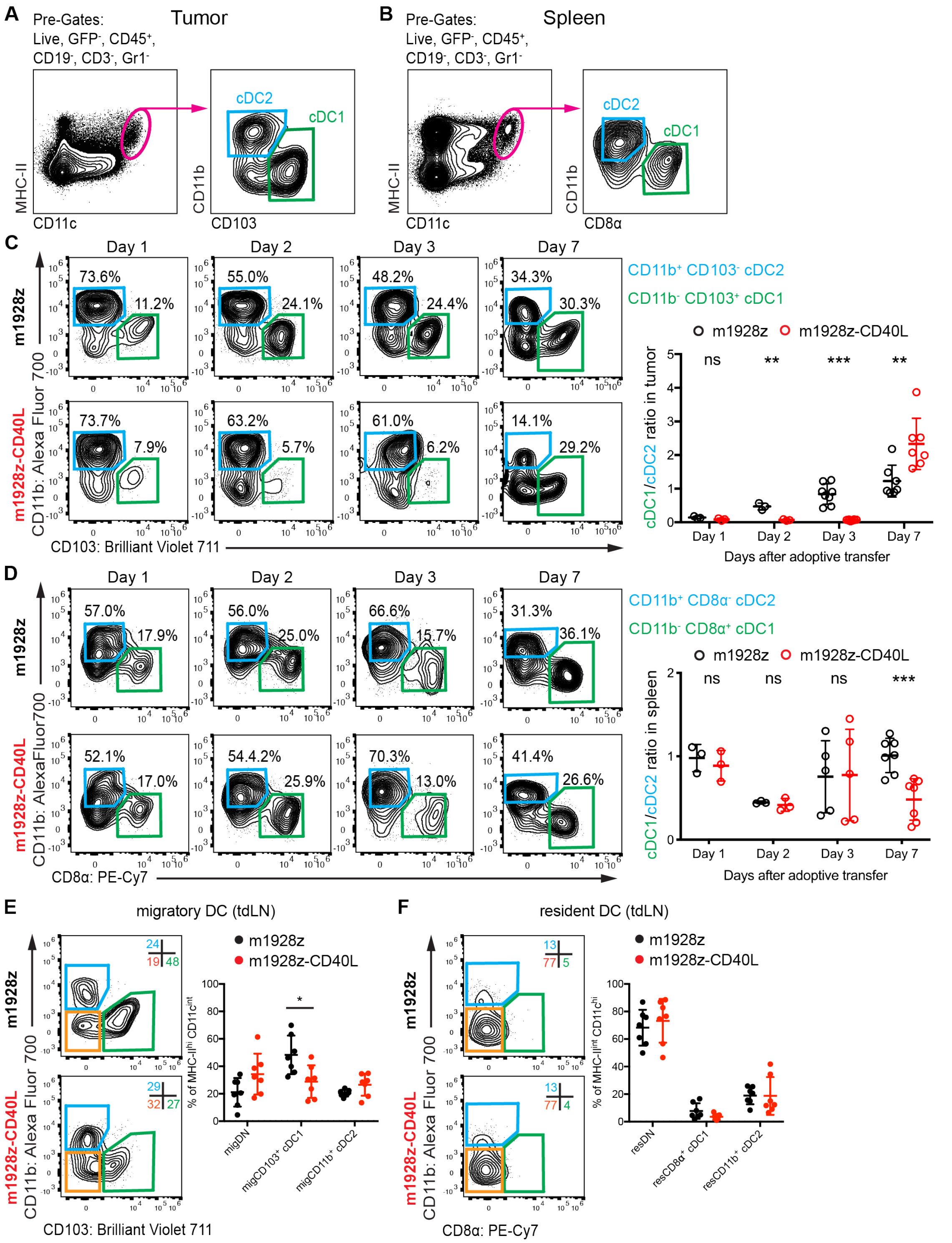

Supplementary Figure 1. m1928z-CD40L CAR T cells increase cDC1/cDC2 ratio in tumor but not in spleen and tumor-draining lymph nodes

a and **b** Gating strategy for MHC-II^{+}CD11c^{+} (pink) cDC1 (green) and cDC2 (blue) populations in tumor (**a**) and spleen (**b**).

c and **d** BALB/c mice were injected with 1x10^{6} A20.GL cells intravenously (i.v.) on day -7 and treated with 3x10^{6} CAR T cells on day 0. Representative flow cytometry contour plots (**c**) on indicated days are shown in the tumor (**c**) and spleen (**d**) of m1928z (top) or m1928z-CD40L

(bottom) CAR T cell-treated mice. Ratios of cDC1/cDC2 populations in the tumor (**c**) and spleen (**d**) are plotted over time. Data in is plotted as mean ± SD (Day 1, 2: n=3/group; Day 3: n=8/group; Day 7: n=7/group).) and results are representative of 2 to 3 experiments. Each dot represents one mouse and p-values were obtained from an unpaired two-tailed Student's t test).
e A20.GL tumor-bearing mice received $3x10^6$ CAR T cells i.v. and the percentage of MHC-II^{hi} $CD11c^{int}$ migratory $CD11b^-CD103^-$ DN (orange), $CD11b^-CD103^+$ cDC1, and $CD11b^+CD103^+$ cDC2 populations in the tumor-draining lymph node (tdLN) was analyzed on day 7.
f A20.GL tumor-bearing mice received $3x10^6$ CAR T cells i.v. and the percentage of MHC-$II^{int}CD11c^{hi}$ resident $CD11b^-CD8\alpha^-$ DN, $CD11b^-CD8\alpha^+$ cDC1, and $CD11b^+CD8\alpha^-$ cDC2 populations in the tdLN was analyzed on day 7.
Data in **e** and **f** is plotted as mean ± SD and pooled from two independent experiments. Each dot represents one mouse (n=7/group) and p-values were obtained from an unpaired two-tailed Student's t test.
Source data are provided as a Source Data file.

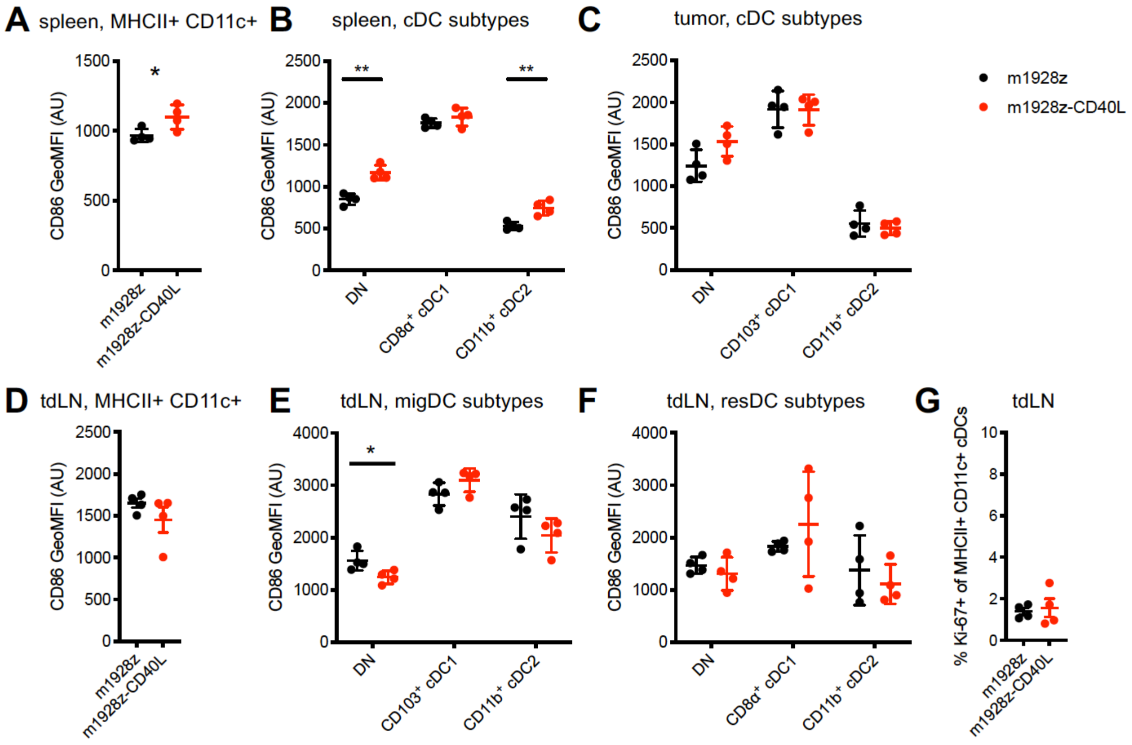

Supplementary Figure 2. cDC1s in different tissues express highest levels of CD86 co-stimulatory molecule among cDCs

a CD86 surface expression on splenic MHCII^{+}CD11c^{+} cDCs on day 7 after CAR T cell treatment in A20.GL tumor-bearing mice.

b and **c** CD86 surface expression on DN, cDC1, and cDC2 subsets on day 7 after CAR T cell treatment in A20.GL tumor-bearing mice in spleen (**b**) and tumor (**c**).

d CD86 surface expression on MHCII^{+}CD11c^{+} cDCs in the tumor-draining lymph node (tdLN) on day 7 after CAR T cell treatment in A20.GL tumor-bearing mice.

e and **f** CD86 surface expression on DN, cDC1, and cDC2 subsets on day 7 after CAR T cell treatment in A20.GL tumor-bearing mice in the tumor-draining lymph node (tdLN) on migratory DCs (**e**) and resident DCs (**f**).

g Percent of Ki-67^{+} cells of all MHCII^{+}CD11c^{+} cDCs in the tdLN on day 7 after T cell treatment in A20.GL tumor-bearing mice.

Data is plotted as mean ± SD and representative of 2-3 independent experiments. Each dot represents one mouse (n=4/group) and p-values were obtained from an unpaired two-tailed Student's t test.

Source data are provided as a Source Data file.

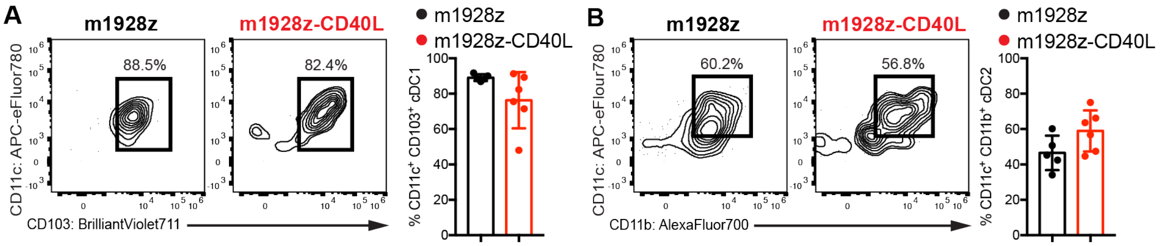

Supplementary Figure 3. In vitro differentiation of tumor-derived cDC1 and cDC2 populations after CAR T cell treatment

a and **b** CD45.2$^+$ A20.GL tumor-bearing mice were treated with 3x10^6 CAR T cells i.v. and CD45.2$^+$CD11b$^-$CD103$^+$ cDC1s (**a**) and CD45.2$^+$CD11b$^+$CD103$^-$ cDC2s (**b**) were isolated from the tumor on day 3 by FACS. Sorted CD45.2$^+$ cDC1s or cDC2s cells were cultured in vitro on a CD45.1 bone-marrow stromal layer for 3 days and the percentage of CD11c$^+$CD103$^+$ cDC1s (**a**) or CD11c$^+$CD11b$^+$ cDC2s of all CD45.2$^+$ cells (**b**) was analyzed. Shown are representative contour plots and the quantification of the percentage of double-positive cells. Data is plotted as mean ± SD and represents in vitro cultures from two mice collected from two independently performed experiments. Each dot represents one in vitro culture (m1928z, n=5; m1928z-CD40L, n=6).

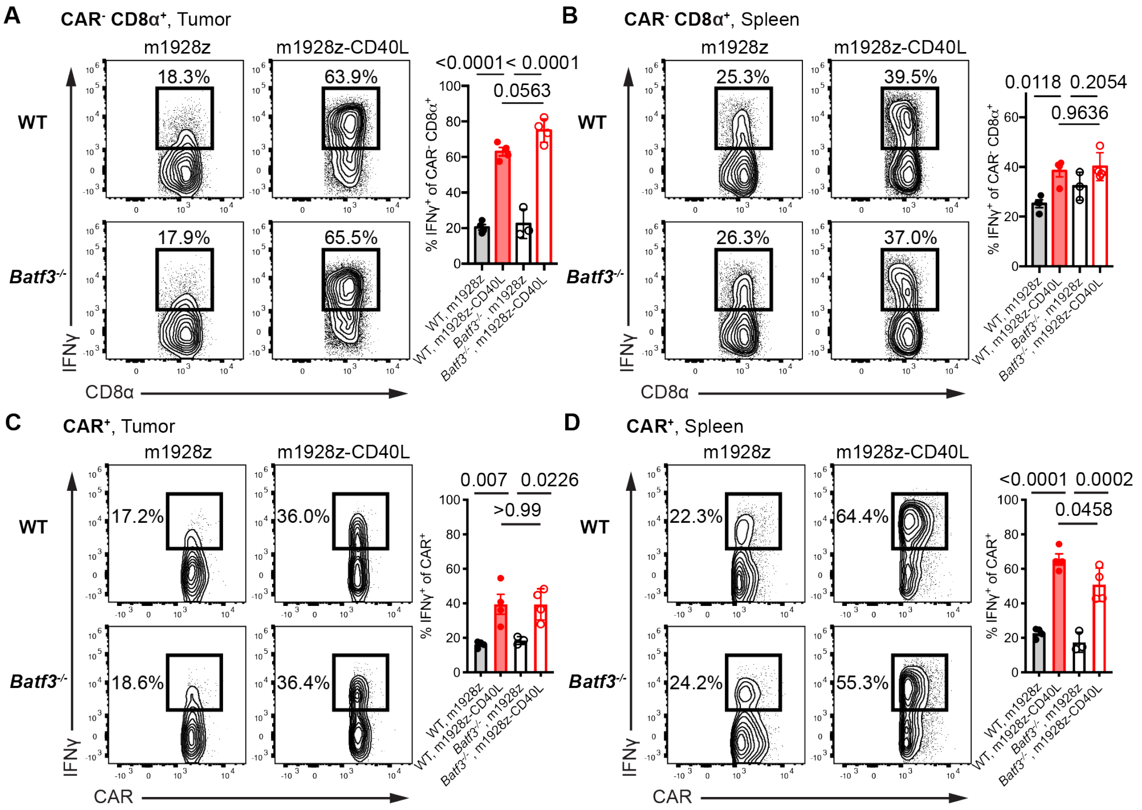

Supplementary Figure 4. Lack of cDC1 population does not affect IFNγ production of endogenous CD8⁺ T cells or adoptively transferred CAR T cells

BALB/c mice were injected intravenously (i.v.) with 1×10^6 A20.GL cells followed by adoptive cell transfer (ACT) of 3×10^6 CAR T cells 7 days after tumor challenge. The tumor and spleens were analyzed 7 days after ACT.

a Flow cytometry contour plots of CAR^-CD8^+ T cells ($CD45^+CD19^-CD11b^-Gr\text{-}1^-CD3^+CAR^-$ pre-gates) after 4 hours ex vivo PMA/ionomycin stimulation isolated from tumor (**a**) and spleen (**b**) of WT (top) or *Batf3*$^{-/-}$ (bottom) mice. IFNγ-producing cells are highlighted by boxed in region. Percentage of $IFN\gamma^+$ cell is summarized on the right.

c and **d** Same as in (**a** and **b**), except that the CAR^+ T cells were analyzed for IFNγ-production. Percentage of $IFN\gamma^+$ cells is summarized on the right. Each dot represents one mouse (WT m1928z, n=4; WT m1928z-CD40L, n=4; *Batf3*$^{-/-}$ m1928z, n=3; *Batf3*$^{-/-}$ m1928z-CD40L, n=4) and data is plotted as mean ± SD. p-values were determined by two-way ANOVA test.

Source data are provided as a Source Data file.

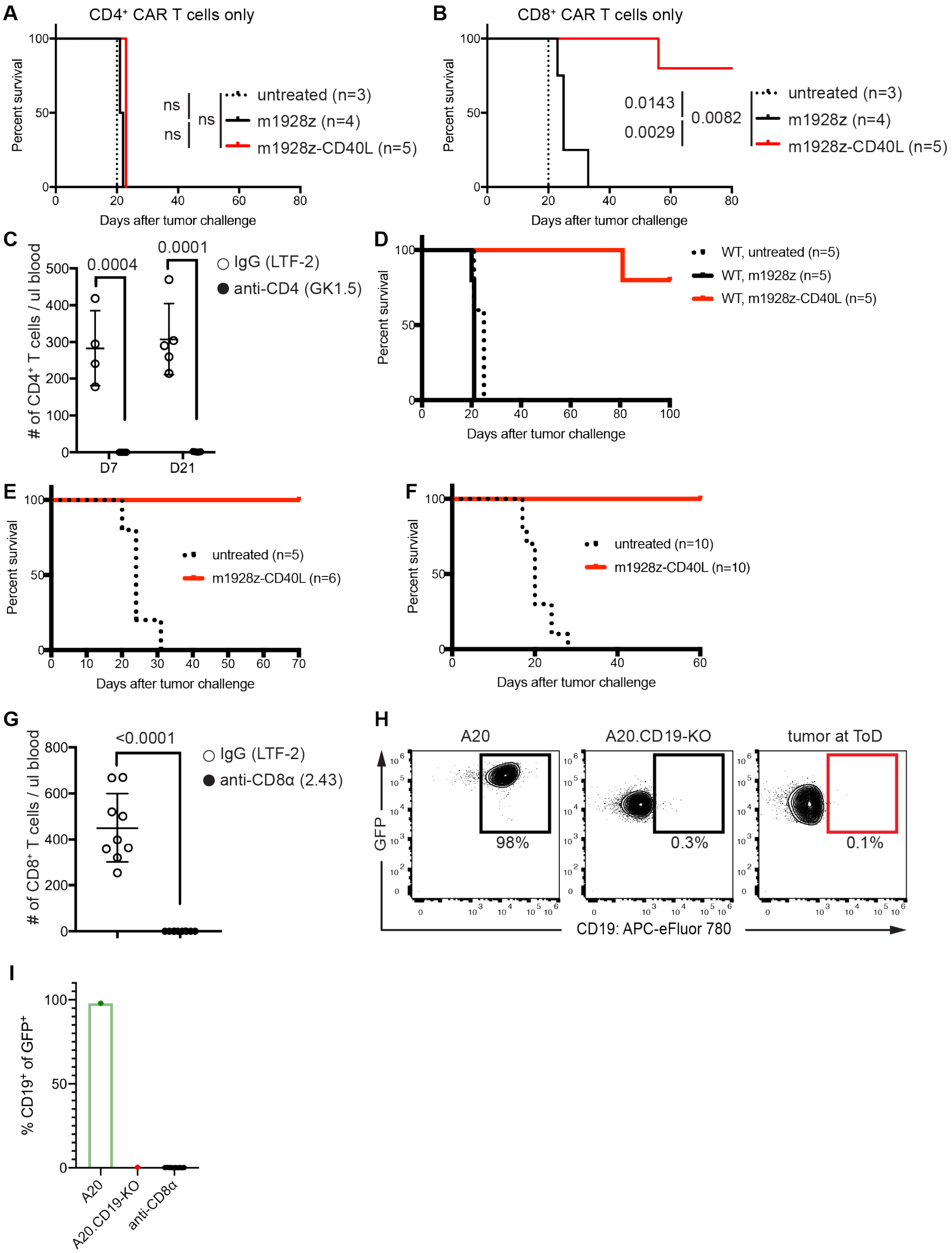

Supplementary Figure 5. CD4⁺ or CD8⁺ CAR T cell treatment and CD4⁺ or CD8⁺ T cell depletion

a and **b** Survival of BALB/c mice injected with 1x10^6 A20.GL tumor cells i.v. on day 0 and treated with 3x10^6 $CD4^+$ (**a**) or $CD8^+$ (**b**) CAR T cells i.v. on day 7. p-values were determined by a two-tailed log-rank (Mantel-Cox) test. ns, non-significant.

c Peripheral blood of m1928z-CD40L CAR T cell treated mice was collected retroorbitally and absolute numbers of $CD4^+$ T cells are plotted at time of adoptive cell transfer (D7) and two weeks later (D21). Mice were either treated with the IgG control antibody (LTF-2) or the CD4-depletion antibody (GK1.5). Each dot represents one mouse (D7 IgG, n=4; others, n=5/group) and data is plotted as mean ± SD.
d, **e**, and **f** Survival of BALB/c mice injected with $1x10^6$ A20.GL tumor cells i.v. on day 0 and treated with $1\text{-}3x10^6$ m1928z-CD40L CAR T cells i.v. on day 7. Three independent experiments are plotted in (**d**-**f**).
g Peripheral blood of long-term surviving mice day 15 after $CD8^+$ T cell depletion was collected retroorbitally and absolute numbers of $CD8^+$ T cells are plotted. Mice were either treated with the IgG control antibody (LTF-2) or the CD8-depletion antibody (2.43). Each dot represents one mouse (IgG/LTF-2, n=9; anti-CD8α/2.43, n=10) and data is plotted as mean ± SD. p-values in (**c**) and (**d**) were obtained from an unpaired two-tailed Student's t-test.
h Surface CD19 expression on GFP^+ tumor cells at time-of-death (ToD) analyzed by flow cytometry and shown for one representative mouse in the $CD8^+$ T cell depleted cohort.
i Summary of CD19 expression on GFP^+ tumor cells (black) of all mice at ToD in the $CD8^+$ T cell depleted cohort. All mice had tumors that were CD19-negative at ToD. CD19 levels of the A20.GL cell line (green) and A20.CD19-KO cell line (red) are plotted as a reference.
Source data are provided as a Source Data file.

Supplementary Tables

Supplementary Table 1: Antibodies, reagents, assays, and software.

REAGENT or RESOURCE	SOURCE	IDENTIFIER
Antibodies		
TruStain fcX (anti-mouse CD16/32)	BioLegend	Cat# 101319, RRID:AB_1574973
anti-mouse CCR7 (clone 4B12) PE	BioLegend	120105
anti-mouse CD3ε (clone 145-2C11) PE-eFluor 610	eBioscience	61-0031, RRID:AB_2574514
anti-mouse CD4 (GK1.5) AlexaFluor 700	eBioscience	56-0041, RRID:AB_493999
anti-mouse CD8α (53-6.7) APC-eFluor 780	eBioscience	47-0081, RRID:AB_1272185
anti-mouse/human CD11b (M1/70) AlexaFluor 700	eBioscience	56-0112, RRID:AB_657585)
anti-mouse CD11c (N418) APC-eFluor 780	eBioscience	47-0114, RRID:AB_1548663
anti-mouse CD19 (eBio1D3) APC-eFluor 780	eBioscience	47-0193, RRID:AB_10853189
anti-mouse CD19 (eBio1D3) PE	eBioscience	12-0193, RRID:AB_657661
anti-mouse CD19 (eBio1D3) PE-eFluor 610	eBioscience	61-0193, RRID:AB_2574536
anti-mouse CD40 (1C10) PerCP-eFluor 710	eBioscience	46-0401, RRID:AB_2573677
anti-mouse CD40L (MR1) PE	eBioscience	12-1541, RRID:AB_465887
anti-mouse CD45 (30-F11) BV605	BioLegend	103139, RRID:AB_2562341
anti-mouse CD45 (30-F11) PE-Cy7	eBioscience	25-0451, RRID:AB_469625
anti-mouse CD45.1 (A20) PE-eFluor610	eBioscience	61-0453, RRID:AB_2574560
anti-mouse CD45.2 (104) PE-Cy7	eBioscience	25-0454, RRID:AB_2573350
anti-mouse CD103 (2E7) BV711	BioLegend	121435, RRID:AB_2686970
anti-mouse IFNγ (XMG1.2) PE-Cy7	eBioscience	25-7311, RRID:AB_1257211
anti-moue IRF8 (V3GYWCH) PerCP-eFluor710	eBioscience	46-9852
anti-mouse Ki-67 (SolA15) PE-eFluor610	eBioscience	61-5698

anti-mouse Ly-6G/Ly-6C (Gr-1) (RB6-8C5) PE-eFluor 610	eBioscience	61-5931, RRID:AB_2574639
anti-mouse Ly-6G/Ly-6C (Gr-1) (RB6-8C5) PE-Cy7	eBioscience	25-5931, RRID:AB_469662
anti-mouse MHC class II (MHC-II) I-A/I-E (M5/114.15.2) BV510	BioLegend	107635, RRID:AB_2561397
anti-human Myc-tag (9B11) AlexaFluor 647	Cell Signaling	2233S, RRID:AB_10693328
anti-mouse NKp46 (29A1.4)	eBioscience	46-3351, RRID:AB_1834442
anti-mouse CD4 (GK1.5)	BioXCell	BE0003-1, RRID:AB_1107636
anti-mouse CD8α (2.43)	BioXCell	BE0061
Chemicals, Peptides, and Recombinant Proteins		
Cell Stimulation Cocktail (plus Protein Transport Inhibitors)	eBioscience	00-4975
LIVE/DEAD Fixable Violet Dead Cell Stain Kit	Thermo Fisher	L34955
RetroNectin Recobminant Human Fibronectin Fragment	Takara	T100B
D-Luciferin, Potassium Salt (Proven and Published)	Gold Biotechnology	LUCK-1G
Recombinant murine Flt3-Ligand	Peprotech	250-31L
Recombinant murine GM-CSF	Peprotech	315-03
Recombinant human IL-2 (Proleukin/Aldesleukin)	Prometheus Therapeutics & Diagnostics	NDC 65483-116-07
Critical Commercial Assays		
EasySep™ Mouse T Cell Isolation Kit	Stemcell Technologies	19851
Fixation/Permeabilization Solution Kit	BD Biosciences	554714
123count eBeads Counting Beads	eBioscience	01-1234-42
Experimental Models: Cell Lines		
Human: Phoenix-ECO	ATCC	CRL-3214, RRID:CVCL_H717
Mouse: A20 lymphoma cell line	ATCC	TIB-208, RRID:CVCL_1940
Mouse: A20.GL	Kuhn et al., 2019	N/A
Mouse: A20.CD19-KO	Kuhn et al., 2019	N/A

Experimental Models: Organisms/Strains		
Mouse: BALB/cAnN	Charles Rivers	CR: 028; RRID:MGI:5654849
Mouse: CBy.PL(B6)-*Thy1*^{a}/ScrJ	The Jackson Laboratory	JAX: 005443; RRID:IMSR_JAX:005443
Mouse: C.129S-*Batf3*^{tm1Kmm}/J	The Jackson Laboratory	JAX: 013756; RRID:IMSR_JAX:013756
Recombinant DNA		
Plasmid: SFG	This study	N/A
Software and Algorithms		
GraphPad Prism v7	GraphPad	https://www.graphpad.com/scientific-software/prism/
FlowJo Version 10	FlowJo LLC	https://www.flowjo.com/
Living Image v2.60	PerkinElmer	https://www.perkinelmer.com/product/spectrum-200-living-image-v4series-1-128113
