# Supplementary material for: CD103+ cDC1 and endogenous CD8+ T cells are necessary for improved CD40L-overexpressing CAR T cell antitumor function
Source: Nat Commun. 2020 Dec 2;11:6171. doi: 10.1038/s41467-020-19833-3 (PMC7710757; doi:10.1038/s41467-020-19833-3)
Supplement: Supplementary file 3 — Reporting Summary [file 41467_2020_19833_MOESM3_ESM.pdf]

nature research

Corresponding author(s): Renier J. Brentjens
Last updated by author(s): Oct 4, 2020

Reporting Summary

Nature Research wishes to improve the reproducibility of the work that we publish. This form provides structure for consistency and transparency in reporting. For further information on Nature Research policies, see Authors & Referees and the Editorial Policy Checklist .

Statistics

For all statistical analyses, confirm that the following items are present in the figure legend, table legend, main text, or Methods section.

n/a	Confirmed	
☐	☒	The exact sample size (*n*) for each experimental group/condition, given as a discrete number and unit of measurement
☐	☒	A statement on whether measurements were taken from distinct samples or whether the same sample was measured repeatedly
☐	☒	The statistical test(s) used AND whether they are one- or two-sided<br>*Only common tests should be described solely by name; describe more complex techniques in the Methods section.*
☒	☐	A description of all covariates tested
☒	☐	A description of any assumptions or corrections, such as tests of normality and adjustment for multiple comparisons
☐	☒	A full description of the statistical parameters including central tendency (e.g. means) or other basic estimates (e.g. regression coefficient) AND variation (e.g. standard deviation) or associated estimates of uncertainty (e.g. confidence intervals)
☐	☒	For null hypothesis testing, the test statistic (e.g. *F*, *t*, *r*) with confidence intervals, effect sizes, degrees of freedom and *P* value noted<br>*Give P values as exact values whenever suitable.*
☒	☐	For Bayesian analysis, information on the choice of priors and Markov chain Monte Carlo settings
☒	☐	For hierarchical and complex designs, identification of the appropriate level for tests and full reporting of outcomes
☒	☐	Estimates of effect sizes (e.g. Cohen's *d*, Pearson's *r*), indicating how they were calculated

Our web collection on statistics for biologists contains articles on many of the points above.

Software and code

Policy information about availability of computer code

Data collection	No software to report.
Data analysis	FlowJo 10, Living Image V2.60, Graph Pad Prism 7 were used for data analysis and quantification.

For manuscripts utilizing custom algorithms or software that are central to the research but not yet described in published literature, software must be made available to editors/reviewers. We strongly encourage code deposition in a community repository (e.g. GitHub). See the Nature Research guidelines for submitting code & software for further information.

Data

Policy information about availability of data

All manuscripts must include a data availability statement. This statement should provide the following information, where applicable:
- Accession codes, unique identifiers, or web links for publicly available datasets
- A list of figures that have associated raw data
- A description of any restrictions on data availability

The authors declare that all data supporting the findings of this study are available within the paper, its supplementary files, and the source data provided with the paper.

Field-specific reporting

Please select the one below that is the best fit for your research. If you are not sure, read the appropriate sections before making your selection.

☒ Life sciences ☐ Behavioural & social sciences ☐ Ecological, evolutionary & environmental sciences

Life sciences study design

All studies must disclose on these points even when the disclosure is negative.

Sample size	Sample sizes were not predetermined based on statistical methods. Sample sizes were chosen based on preliminary experiments that used similar sample sizes and successfully detected differences between groups.
Data exclusions	No data was excluded.
Replication	Survival experiments in wild-type and Batf3-/- mice were performed 2x independently. Results were pooled. To this end, survival experiments in Figure 4 were not replicated because of a lack of long-term surviving mice.<br><br>Experiments in Figures 1 and 3, and Supplementary Figures 1, 2, and 3 were performed 2x independently. Results were pooled.<br>Experiments in Supplementary Figure 4 requiring wild-type mice were successfully repeated in an independent experiment. Experiments in Supplementary Figure 4 requiring Batf3-/- were not repeated, due to lack of transgenic animals.<br>Experiments in Supplementary Figure 5 were all performed 2x independently.<br><br>All attempts at replication were successful.
Randomization	Tumor-bearing animals were randomized into control and treatment groups before CAR T cell injection, both for survival and non-survival experiments.
Blinding	Investigators injecting CAR T cells were not blinded because same investigators prepared and injected CAR T cells. For assessment of survival and tumor burden by imaging, investigators were blinded to treatment groups. Investigators were not blinded for other experiments because same investigators euthanized, processed, and analyzed the samples.

Reporting for specific materials, systems and methods

We require information from authors about some types of materials, experimental systems and methods used in many studies. Here, indicate whether each material, system or method listed is relevant to your study. If you are not sure if a list item applies to your research, read the appropriate section before selecting a response.

Materials & experimental systems

n/a	Involved in the study	
☐	☒	Antibodies
☐	☒	Eukaryotic cell lines
☒	☐	Palaeontology
☐	☒	Animals and other organisms
☒	☐	Human research participants
☒	☐	Clinical data

Methods

n/a	Involved in the study	
☒	☐	ChIP-seq
☐	☒	Flow cytometry
☒	☐	MRI-based neuroimaging

Antibodies

Antibodies used

TruStain fcX (anti-mouse CD16/32) BioLegend Cat# 101319, RRID:AB_1574973, 5 µg/ml
anti-mouse CCR7 (clone 4B12) PE BioLegend, 120105, 2 µg/ml
anti-mouse CD3 (clone 17A2) BrilliantViolet510 BioLegend 100233, RRID:AB_2561387, 1 µg/ml
anti-mouse CD3ε (clone 145-2C11) PE-eFluor 610 eBioscience 61-0031, RRID:AB_2574514, 1 µg/ml
anti-mouse CD4 (GK1.5) AlexaFluor 700 eBioscience 56-0041, RRID:AB_493999, 0.1 µg/ml
anti-mouse CD8α (53-6.7) APC-eFluor 780 eBioscience 47-0081, RRID:AB_1272185, 0.1 µg/ml
anti-mouse/human CD11b (M1/70) AlexaFluor 700 eBioscience 56-0112, RRID:AB_657585), 0.1 µg/ml
anti-mouse CD11c (N418) APC-eFluor 780 eBioscience 47-0114, RRID:AB_1548663, 0.2 µg/ml
anti-mouse CD19 (eBio1D3) APC-eFluor 780 eBioscience 47-0193, RRID:AB_10853189, 0.1 µg/ml
anti-mouse CD19 (eBio1D3) PE eBioscience 12-0193, RRID:AB_657661, 0.1 µg/ml
anti-mouse CD19 (eBio1D3) PE-eFluor 610 eBioscience 61-0193, RRID:AB_2574536, 0.5 µg/ml
anti-mouse CD40 (1C10) PerCP-eFluor 710 eBioscience 46-0401, RRID:AB_2573677, 1 µg/ml
anti-mouse CD40L (MR1) PE eBioscience 12-1541, RRID:AB_465887, 0.2 µg/ml
anti-mouse CD45 (30-F11) BV605 BioLegend 103139, RRID:AB_2562341, 0.5 µg/ml
anti-mouse CD45 (30-F11) PE-Cy7 eBioscience 25-0451, RRID:AB_469625, 0.5 µg/ml
anti-mouse CD45.1 (A20) PE-eFluor610 eBioscience 61-0453, RRID:AB_2574560, 0.2 µg/ml
anti-mouse CD45.2 (104) PE-Cy7 eBioscience 25-0454, RRID:AB_2573350, 0.1 µg/ml
anti-mouse CD103 (2E7) BV711 BioLegend 121435, RRID:AB_2686970, 1 µg/ml

	anti-mouse IFNγ (XMG1.2) PE-Cy7 eBioscience 25-7311, RRID:AB_1257211, 0.4 µg/ml<br>anti-moue IRF8 (V3GYWCH) PerCP-eFluor710 eBioscience 46-9852, 1.6 µg/ml<br>anti-mouse Ki-67 (SolA15) PE-eFluor610 eBioscience 61-5698, 0.1 µg/ml<br>anti-mouse Ly-6G/Ly-6C (Gr-1) (RB6-8C5) PE-eFluor 610 eBioscience 61-5931, RRID:AB_2574639, 0.2 µg/ml<br>anti-mouse Ly-6G/Ly-6C (Gr-1) (RB6-8C5) PE-Cy7 eBioscience 25-5931, RRID:AB_469662, 0.2 µg/ml<br>anti-mouse MHC class II (MHC-II) I-A/I-E (M5/114.15.2) BV510 BioLegend 107635, RRID:AB_2561397, 0.2 µg/ml<br>anti-human Myc-tag (9B11) AlexaFluor 647 Cell Signaling 2233S, RRID:AB_10693328, 1:500<br>anti-mouse CD4 (GK1.5) BioXCell BE0003-1, RRID:AB_1107636, 200 µg<br>anti-mouse CD8α (2.43) BioXCell BE0061, RRID:AB_1125541, 200 µg
Validation	Each fluorophore-conjugated antibody used in flow cytometric experiments was titrated via serial dilutions and using marker positive and marker negative cells. Each cell-depleting antibody was tested in naive mice prior to use in experiments. All used antibodies are commercially available and validated per manufacturer's website.

Eukaryotic cell lines

Policy information about cell lines

Cell line source(s)	A20 and Phoenix-ECO cells were obtained from ATCC.
Authentication	Certificate of authentication was received from ATCC. No further authentication was performed.
Mycoplasma contamination	Used cell lines were tested for mycoplasma contamination and always found to be negative.
Commonly misidentified lines<br>(See ICLAC register)	No commonly misidentified cell line per ICLAC register was used.

Animals and other organisms

Policy information about studies involving animals; ARRIVE guidelines recommended for reporting animal research

Laboratory animals	All mice were housed in pathogen-free conditions and kept in a room with controlled temperature (~22 degrees C) and humidity under 12 h light/dark cycle.<br><br>8-12 week old female wild-type BALB/cAnN<br>8-12 week old female BALB/c CD45.1 (CByJ.SJL(B6)-Ptprca/J)<br>8-12 week old female and male BALB/c CD40-/- (CNCr.129P2-Cd40tm1Kik/J)<br>8-12 week old female BALB/c Batf3-/- (C.129S-Batf3tm1Kmm/J)<br>For the experiment in Figure 4, 15-36 week old female wild-type BALB/cAnN mice were used as age-matched controls.
Wild animals	This study did not involve wild animals.
Field-collected samples	This study did not involve samples collected in the field.
Ethics oversight	All experiments were performed in accordance with the MSKCC Institutional Animal Care and Use Committee (IACUC) approved protocol guidelines (MSKCC #00-05-065)

Note that full information on the approval of the study protocol must also be provided in the manuscript.

Flow Cytometry

Plots

Confirm that:

- [x] The axis labels state the marker and fluorochrome used (e.g. CD4-FITC).
- [x] The axis scales are clearly visible. Include numbers along axes only for bottom left plot of group (a 'group' is an analysis of identical markers).
- [x] All plots are contour plots with outliers or pseudocolor plots.
- [x] A numerical value for number of cells or percentage (with statistics) is provided.

Methodology

Sample preparation	Mouse T cells were processed as described previously6. Briefly, murine T cells were isolated from spleens of euthanized mice via negative selection using the EasySep Mouse T cell Isolation Kit (StemCell). Cells were then expanded in RPMI-1640 supplemented with 10% heat-inactivated FBS, nonessential amino acids, 1 mM sodium pyruvate, 10 mM HEPES, 2 mM L-glutamine, 1% penicillin/streptomycin, 11 mM glucose, 2 µM 2-mercaptoethanol, 100 IU of recombinant human IL-2 (rhIL-2) (Prometheus Therapeutics & Diagnostics), and stimulated with anti-CD3/28 Dynabeads (Life Technologies) at a ratio of 1-to-2 (bead-to-cell).

24 and 48 hr after bead stimulation, T cells were spinoculated on retronectin-coated plates with viral supernatant collected from Phoenix-ECO cells. After the second spinoculation, cells were rested for one day and then used in subsequent experiments.

Mice were euthanized via CO2 inhalation prior to organ removal. Harvested spleens were minced, filtered, washed in PBS, and red blood cells were lysed. Tumor tissue from the liver was mechanically disrupted, filtered, separated by Percoll density centrifugation, and red blood cells were lysed. Remaining cells were washed in PBS, counted, and used in subsequent analyses.

Instrument	Beckman Coulter Gallios, ThermoFisher Attune NxT, BD FACSAria, IVIS SpectrumCT imaging system
Software	FlowJo 10 (Treestar)
Cell population abundance	100,000+ cells per cell type of interest were sorted. Purity of cell populations was determined by reanalysis of an aliquot of sorted cell samples.
Gating strategy	SSC-A/FSC-A parameters were used to gate out debris. SSC/FSC height and width parameters were used to exclude doublets. Live singlets were then identified using DAPI or other fluorescent LIVE/DEAD stain.<br><br>Negative-control (unstained and Flow-minus-One), marker-negative and fluorophore/marker-positive cells were used to establish gates for each cell type.<br><br>Gating strategy for cDC1/cDC2 identification is described in Supplementary Figure 1.<br>Gating strategy for migratory and resident DCs is depicted in Figure 1D.

☒ Tick this box to confirm that a figure exemplifying the gating strategy is provided in the Supplementary Information.
